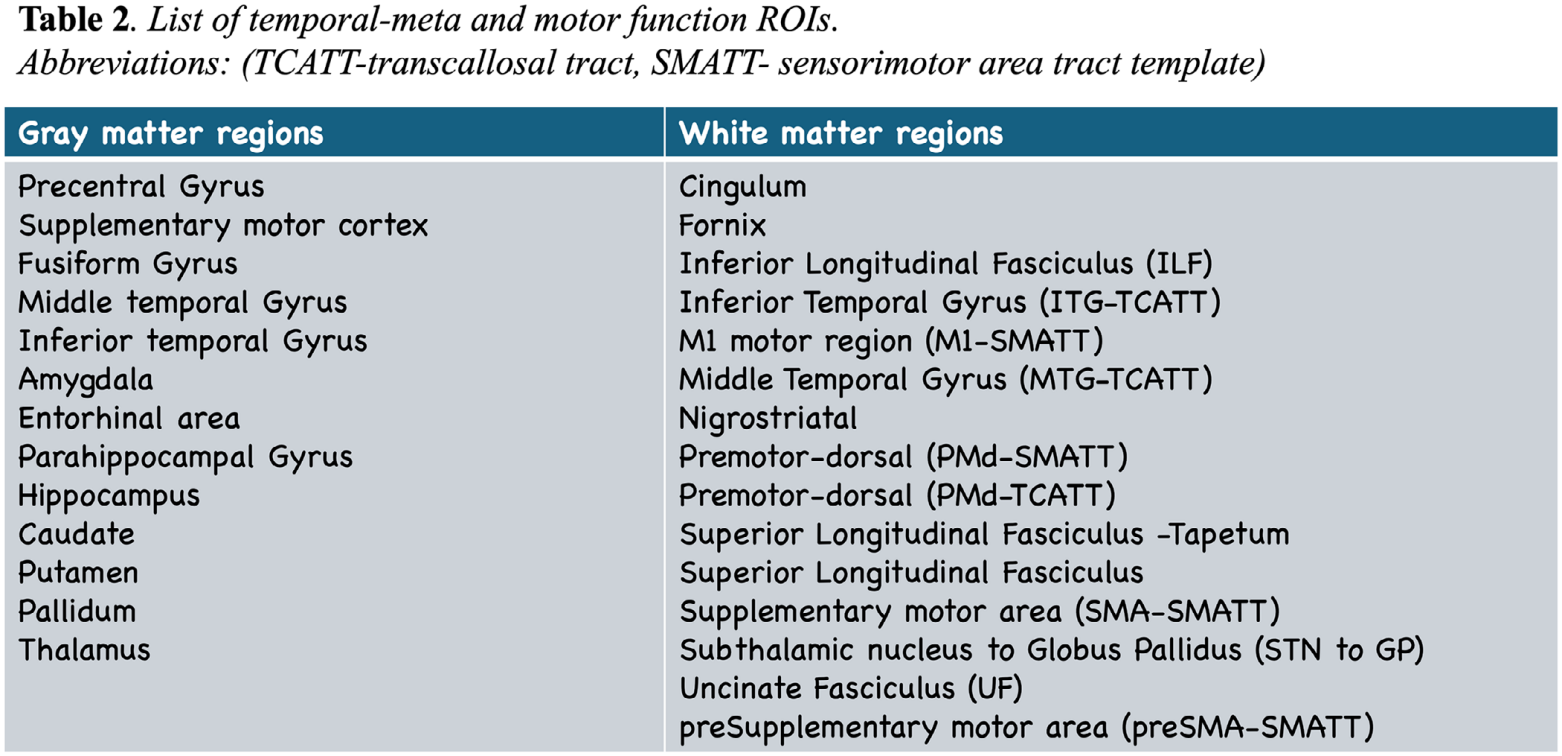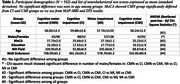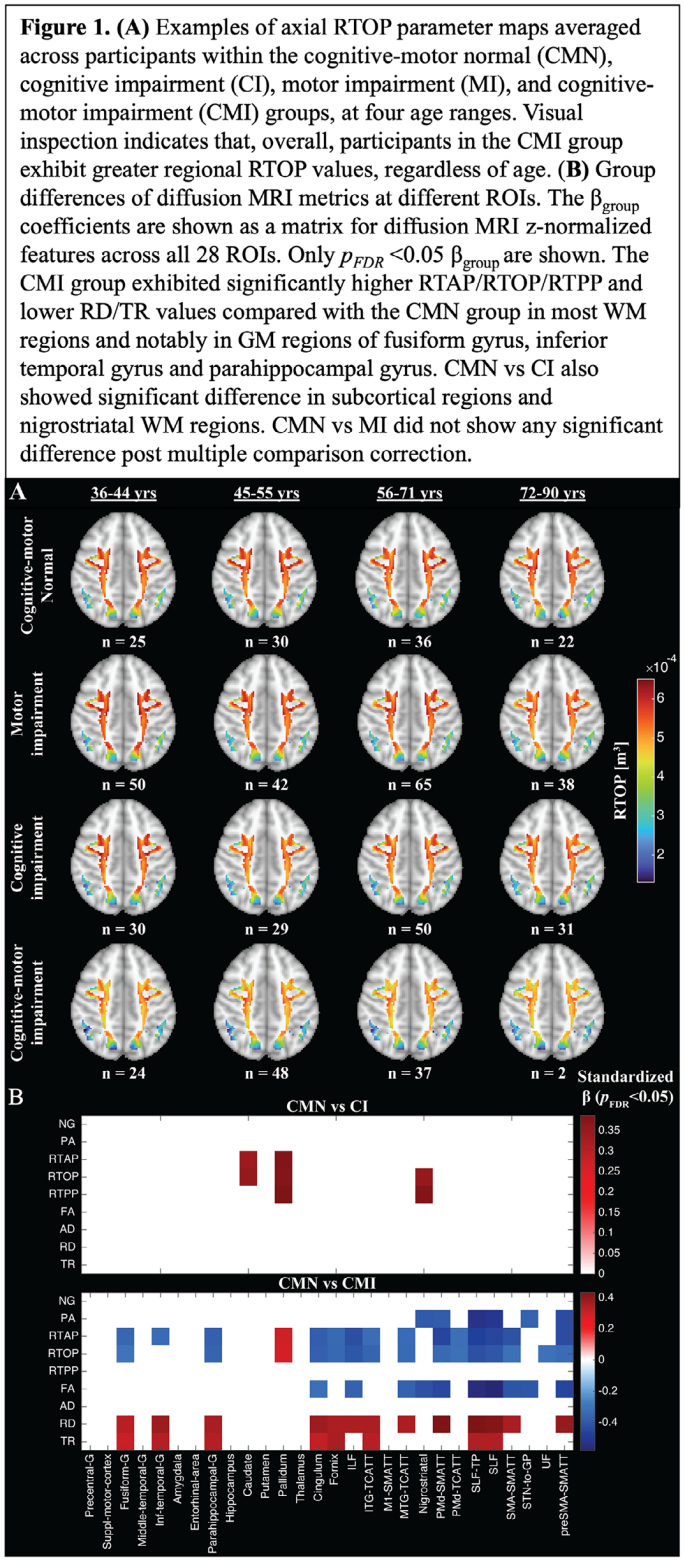# Cognitive‐motor impairment in HCP‐Aging cohort and its relation to gray and white matter microstructural changes

**DOI:** 10.1002/alz70856_103623

**Published:** 2025-12-24

**Authors:** Kavita Singh, Kurt Schilling, Yang An, Dan Benjamini

**Affiliations:** ^1^ Multiscale Imaging and Integrative Biophysics Unit, National Institute on Aging, NIH, Baltimore, MD, USA; ^2^ Department of Radiology & Radiological Sciences, Vanderbilt University Medical Center, Nashville, TN, USA; ^3^ Laboratory of Behavioral Neuroscience, National Institute on Aging, Intramural Research Program, Baltimore, MD, USA

## Abstract

**Background:**

40% of individuals aged over 65 years suffer memory loss with increasing evidence showing early or concomitant motor decline which might be warning sign for neurodegenerative diseases like Alzheimer's disease (AD). Gray matter (GM) temporal‐meta regions (ROIs) are affected early in aging/AD population, but little work has been done in conjunction with motor‐related regions. Current work (a) characterized cognitive and motor impairment in aging cohort; (b) assessed GM and WM microstructural changes using the mean apparent propagator (MAP) diffusion MRI model along with DTI; and (c) validated findings with standard MoCA screening.

**Method:**

707 subjects (36 to 90 years) imaging dataset from Human Connectome Project‐Aging (HCP‐A) was used. NIH‐Fluid and walk‐endurance measures classified cognitive‐motor normal (CMN), cognitive impairment (CI), motor impairment (MI) and cognitive‐motor impairment (CMI) age‐matched groups using k‐means clustering. Five MAP‐MRI metrics (return to origin/axis/plane probability‐RTOP, RTAP, RTPP, non‐Gaussianity‐NG, and propagator anisotropy‐PA) and 4 DTI metrics (fractional anisotropy‐FA, axial diffusivity‐AD, radial diffusivity‐RD, and trace‐TR) were examined. Anatomical images were used to segment GM and WM tract regions that are related to temporal‐meta ROIs and motor function (Table 2). Linear regression model assessed GM and WM differences between the 4 groups: 𝑃𝑖=𝛽0+𝛽_group_*group+𝛽_sex_*𝑠𝑒𝑥+𝛽_YOE_*𝑌𝑂𝐸+𝛽_site_*𝑠𝑖𝑡𝑒+𝛽_BMI_*BMI, (𝑃𝑖 ‐ mean MRI ROI value for the *i*th ROI). Sex, years of education (YOE), body mass index (BMI) and site were co‐variates. False discovery rate corrected *p*‐value < 0.05 was used.

**Result:**

Table 1 shows participant demographics and neurobehavioral test results. Examples of axial RTOP maps averaged across participants from the 4 groups at different age ranges are shown in Figure 1A. These maps indicate that the CMI group exhibits greater regional RTOP values, regardless of age. Regression results, shown in Figure 1B, reveal significantly higher RTAP/RTOP/RTPP and lower RD/TR values in the CMI group, compared with the CMN group in regions with overlapping cognitive and motor functions and motor specific regions. CMN compared to CI showed significant inverse results in caudate, pallidum and nigrostriatal labels. CMN compared to MI showed no significant difference.

**Conclusion:**

Concomitant motor and cognitive impairment lead to enhanced brain GM and WM changes in aging population.